# Case of a Curvature of the Spine Successfully Treated

**Published:** 1785

**Authors:** 


### IV. *Case of a Curvature of the Spine successfully treated.* *By Mr.* J. Munning, *Surgeon, in London.*

A Man, forty-seven years old, whose employment was that of a porter, began, about the year 1779, to complain of a soreness and stiffness of his neck and upper part of his back. To these symptoms, which he attributed to his having carried a heavy load on his head, at the end of ten or twelve months, was added a similar sensation in his loins, together with a slight degree of inability to move his lower extremities.

Two years after this appeared a projection of the upper part of the spine, but attended with no pain in that part, (a circumstance peculiar to this disease); and he was now seized, at times, with lassitude and spasms; frequently stumbled, and sometimes fell down. All these symptoms and appearances continued gradually to increase during five years, at the end of which period he applied to me.

He was then much emaciated, with his head bent forward, so that his chin rested on the sternum; and he had at times, and in different parts of his body, violent spasms, insomuch, that that the mufcles of his arms and hands were fo rigid and diftended, that he could not bend his fingers. His organs of refpiration were equally affected, fo that his breathing was rendered extremely laborious, attended with a quick and contracted pulfe. He expectorated a great deal of phlegm; had little or no appetite; and was truly in a hectic ftate. He was alfo much troubled with heat of urine, and was of a coftive habit of body.

On examining the fpine, the two laft cervical vertebræ, and the two firft dorfal vertebræ, were found to be greatly diftorted, forming nearly a femicircle. A crepitus and fluctuation were evident; and, from what I have feen in a variety of diffections in this difeafe, of fubjects who have died without the application of the cauftic, I have no doubt that the bodies of the vertebræ were, in this patient, in a truly carious ftate, and that matter was formed in them, fo that the difeafe muft, in a fhort time, have terminated fatally, had not a cure been attempted. This was done by cauftics, according to the method defcribed by Mr. Pott in his excellent publication on this fubject.

In three days after the application of the cauftics there was evidently a diminution of the fpafmodic

ſpaſmodic contractions; and as they continued to diſcharge freely, every bad ſymptom gradually leſſened. In three months he found himſelf ſo much relieved, that the iſſues were ſuffered nearly to heal; but the ſymptoms then beginning to return again, freſh cauſtics were applied, and with the ſame good effect as before. Theſe were kept open four months; at the end of which time he found himſelf well. The curvature, however, ſtill remained, though in a leſs degree than at firſt.

After the iſſues had been healed about a fortnight, he had a ſlight return of his former ſymptoms, which induced me to adviſe another renewal of the cauſtics. Theſe laſt produced a plentiful diſcharge, together with an eryſipelatous inflammation in the vicinity of the curve. I was now determined, if poſſible, to keep them open till I had reduced the whole of the curvature; and in this I ſucceeded in the courſe of ten months. The patient is now upright, and in a perfect ſtate of health.

A more general application of the cauſtics in this diſeaſe is much to be wiſhed for, as it is to be feared that neither medicine, nor any kind of machine, however well it may be con-

ſtructed, will have any good effect in this complaint.

*Falcon Square,*

*Alderſgate Street,*

Aug. 30, 1785.

